# A Cost Effective (QbD) Approach in the Development and Optimization of Rosiglitazone Maleate Mucoadhesive Extended Release Tablets –*In Vitro* and *Ex Vivo*

**DOI:** 10.15171/apb.2019.032

**Published:** 2019-06-01

**Authors:** Suryaprakash Reddy Chappidi, Eranti Bhargav, Venkataranganna Marikunte, Haranath Chinthaginjala, Mallela Vijaya Jyothi, Muralidhar Pisay, Mounika Jutur, Mujahid Shaik Mahammad, Mrunalini Poura, Sailaja Yadav, Moinuddin Syed

**Affiliations:** ^1^RERDS-CPR, Raghavendra Institute of Pharmaceutical Education and Research, Anantapur- 515721.; ^2^Connexios Life Sciences Pvt Ltd, Basavanagudi, Bangalore, 560004.; ^3^Department of Pharmaceutics, Raghavendra Institute of Pharmaceutical Education and Research, Anantapur- 515721.

**Keywords:** Quality by design, Design of experiment, Carbopol 934P, Sodium carboxymethyl cellulose

## Abstract

***Purpose:*** The purpose of the study was to develop and optimize rosiglitazone maleate mucoadhesive extended-release tablets by quality by design (QbD) approach. Based on QTPP (quality target product profile) CQAs (critical quality attributes) were identified.

***Methods:*** Failure mode and effects analysis (FMEA) method were adopted for risk assessment. Risk analysis by the evaluation of formulation and process parameters showed that the optimizing the levels of polymers could reduce high risk to achieve target profile. Drug-excipient compatibility studies by Fourier transforms infra-red and DSC studies showed that the drug was compatible with the polymers used. Design of experiment (DoE) performed by Sigma tech software, Carbopol 934P and sodium carboxymethyl cellulose (SCMC) were identified as independent variables and hardness, drug release at 12 hours and ex vivo mucoadhesion time were adopted as responses. Contour plots generated from the software were used for identification of design space.

***Results:*** Carbopol 934P and SCMC had positive and negative effects respectively on the selected responses. Higher the concentration of Carbopol 934P and lower the concentration of SCMC mucoadhesive extended release criteria could be achieved. Drug release kinetics followed first order release with Higuchi diffusion and Fickian diffusion. Ex vivo mucoadhesion test on goat stomach mucosa indicated that adhesion time increased at higher concentrations of Carbopol 934P. Optimized formula satisfying all the required parameters was selected and evaluated. The predicted response values were in close agreement with experimental response values, confirmed by calculating standard error.

***Conclusion:*** It has been concluded that the application of QbD in the optimization reduced the number of trials to produce a cost-effective formula.

## Introduction

Diabetes mellitus (type 2) occurs due to inadequate production of insulin by the pancreas or may be due to insulin resistance which results in increased blood glucose levels. Around the world, almost 400 million cases have shown diabetes which may be expected to increase around 472 million by 2030.^1^ Several oral antidiabetic formulations were available in the market to treat type 2 diabetes mellitus. In the present study rosiglitazone maleate, a peroxisome proliferator-activated receptor-gamma (PPAR-gamma) agonist which reduces production of hepatic glucose and fat content by improving peripheral insulin sensitivity was selected. Rosiglitazone maleate has shorter half-life about 3-4 hours and to reach peak plasma concentration it takes 1 hour. Further anaemia, oedema and weight gain are frequently reported adverse effects with repeated immediate release dosage forms. It is soluble and absorbed in the stomach especially in the upper parts of the gastrointestinal tract, its bioavailability is greatly reduced as the gastric emptying is faster and reduction of its stay in the stomach.^2^ Hence there is a need to develop an extended release dosage form to reduce frequent administrations which maintain steady-state plasma concentrations and by extending its release in the stomach. Extended-release dosage forms are types of dosage forms that extend the absorption and release of the drug for a prolonged period.^3^ There are several methods for extending the release of the drug. In the present study, mucoadhesive polymers were selected to reduce emptying time and to extend the release of the drug rosiglitazone maleate in the stomach. Apart from an antidiabetic drug, the clinical trials^4^ have shown that rosiglitazone maleate extended-release tablets can be used as an adjunctive therapy for subjects with mild to moderate Alzheimer’s disease. Studies have shown that in the treatment of both acute and chronic leukemia rosiglitazone can provide additional benefit as an extended-release form. In the year 2010 FDA has restricted the use of Avandia (rosiglitazone maleate).^5^ In 2015 FDA has removed certain restrictions on the diabetes drug Avandia. Studies had shown that it did not increase the risk of heart attack and death.^6^ It still has great prospects for markets.^7^

Excipients for extending the release of the drug in the stomach by mucoadhesion are crucial which includes careful selection of flexible, cost-effective and regulatory acceptance of mucoadhesive polymers for the formulation of extended-release tablets.^8^ Quality by design (QbD) implementation in the development of cost-effective formulations reduces the defects and product variability by setting up of quality target product profile (QTPP), process and product design its understanding, risk assessment by design of experiment (DoE), control strategy and continual improvement.^9^ In the present study, an attempt was made to develop a cost-effective method (QbD) for the designing of rosiglitazone maleate Extended-release tablets.

## Materials and Methods

Rosiglitazone maleate was obtained as gift sample from Connexios Life Sciences Pvt Ltd (Banglore, India). Carbopol 934P and sodium carboxymethyl cellulose (SCMC) were purchased from Himedia Laboratories Private Limited, Banglore, India. All ingredients used were of analytical grade.

### Preformulation studies

Organoleptic characters such as appearance, color, odor and melting point were studied for the rosiglitazone maleate.

### Setting up of QTPP for the formulation

It involves setting up of targets and requirements based on reference listed drug which includes but not limited to dosage form, route of administration, strength, drug release or delivery of the drug, pharmacokinetic characteristics, stability.

### Study of CQA of formulation and process

A developed and formulated product meets the requirements such as safety, efficacy performance and stability when the critical quality attribute (CQA) are controlled. Only the quality attributes that are crucial should be identified.

### Excipient compatibility studies

#### Fourier transforms infra-red (FTIR) spectroscopy

FT-IR spectroscopy (FTIR-Bruker model) studies were carried out to find out the compatibility of the drug with the selected excipients. Two milligrams of pure rosiglitazone maleate drug and optimized formulation were dispersed separately in potassium bromide powder and by applying 6 tons pressure the pellets were prepared. Pure drug pellets and optimized pellet formulation were scanned separately. Both the spectra were compared for confirmation of common peaks.

### Differential scanning calorimetry

Accurately 5 mg of rosiglitazone maleate was weighed and sieved through the #60 sieve, transferred to DSC aluminium pan and scanned at 25-200°C temperature at 10°C/min heating rate. The procedure was repeated by placing the blend of the optimized formulation to obtain thermograms. The obtained thermograms were compared to identify for any interaction between them.^10^

### Risk assessment for drug substance attributes

Failure mode and effects analysis (FMEA) method was adopted for the risk analysis by the evaluation of formulation and process parameters. Based on the physicochemical and biological properties of the drug substance the initial risk assessment on drug product CQAs was classified into 3 classes viz low, medium and high.

### Initial risk assessment of formulation variables

For formulation development, an initial risk assessment was made and the manufacturing process has not been established in detail. Based on the above parameters, risks were rated based on assumption that for each formulation attribute that changed, an optimized manufacturing process would be established. Polymer levels (Carbopol 934, SCMC, ethyl cellulose) in the formulation, as well as talc and magnesium stearate levels, are considered as critical variables, hardness, drug release and *ex vivo* mucoadhesion time were considered as process variables and the risk assessment had been discussed.^11^

### Formulation development

The aim was to select a polymer with its level which suits to the target profile (Hardness, Dissolution). Polymers (Carbopol 934, SCMC, ethyl cellulose) were selected after conduction of several trials to match with the targeted variables. Ethyl cellulose is a hydrophobic polymer extended the release but could not match QTPP (extended release criteria). Hence the polymer was not further selected for the studies. Whereas the hydrophilic matrix polymers Carbopol 934 and SCMC showed the hardness and release profiles with the stated QTPP. Talc and magnesium stearate were used as lubricants with hydrophobic nature hence risk assessment was made to study their behavior as expected to target with QTPP.

For determining the polymer concentration levels DoE was selected. Based on DoE the polymers Carbopol 934 and SCMC were selected as independent variables/factors, whereas hardness, % cumulative drug release at 12 h and *ex vivo* mucoadhesion time was selected as dependent variables/responses. 2^2^ factorial design with 4 replicates was selected for formulation design and the model was found to be nonlinear. Hence the study was further extended to central composite design.^12^

Rosiglitazone maleate, Carbapol 934, SCMC and microcrystalline cellulose PH – 102 were weighed and sieved individually through #45. By using mortar and pestle roiglitazone maleate was added and mixed uniformly with Carbapol 934, SCMC, Microcrystalline cellulose by geometric dilution with gentle trituration to get a uniform mixture. Finally, magnesium stearate and talc were added which were previously sieved through #60 and mixed with the above drug blend. All the formulated blend was evaluated for precompression parameters.

### Evaluation of pre-compression parameters

One hundred milliliters graduated cylinder was held in an inclined position and transferred accurately weighed blend of formulation into it. Initial weight and volume were noted. Bulk density = Weight of the sample/volume of the sample.^13^ To determine tapped density a measuring cylinder of 100 mL capacity was taken and an accurately weighed blend of the sample was transferred (Electrolab Tapped Density Apparatus). Initial volume (V_0_) was noted and tapped the cylinder for 10 times, noted the final volume. Continued tapings for 500 times. Since the difference between the volume was more than 2ml after 10 and 500 tapings so further continued for 1250 tapings. Hausner ratio (HR) was determined using the formula

HR = Tapped density/ Bulk density X 100

Compressibility index (CI) was calculated using the formula CI % = Tapped density of the powder - the Bulk density of the powder / Tapped density of the powder X 100.

A funnel was taken and placed vertically to a cone height (h) of the maximum level then poured the amoxicillin blend through it, heap radius (r) was measured. The calculated angle of repose using the formula: tan θ = Cone height / Heap radius. Experimentation was carried out in triplicate.

### Compression of rosiglitazone maleate blend

Rotary tablet compression machine (Rimek) was used for compressing the formulated blend into tablets and evaluated for all the post-compression parameters.

### Evaluation of post-compression parameters

The tablets were inspected for smoothness, the absence of cracks, chips and other undesirable characteristics.^14^ 20 tablets were selected randomly, the weight of the individual tablet and the average weight of the tablets were noted. Thickness was determined using digital vernier calipers for 20 tablets from each batch and expressed the average thickness in mm. Monsanto hardness tester was used to determine the hardness of tablets. 10 tablets hardness was noted and the average hardness was calculated,^15^ expressed in kg/cm^2^. 10 tablets were taken its initial weight was noted and were placed in Roche friabilator rotated for 100 revolutions at 25 rpm and then de-dusted and reweighed. The percentage friability was calculated using the formula:

Percentage friability = {(A-B)/B} × 100

where, A = Initial weight of tablets, B = Final weight of tablets after 100 revolutions

### Drug content

Randomly 20 tablets were selected and crushed. To 8 mg of the drug, the equivalent powder was weighed and transferred to the 100 mL volumetric flask. 80 mL of 0.1 M Hydrochloric acid was added and shaken for 10 minutes using a mechanical shaker. Filtered the solution after making up the volume to 100 mL. 10 ml of the filtrate was collected and diluted to 100 mL with 0.1 M Hydrochloric acid. Further 10 mL of the resulting filtered solution was taken and diluted with 0.1 M Hydrochloric acid to 100 mL. Then the final obtained solution was evaluated to determine drug content of tablets at 313 nm by UV-Visible spectrophotometer (Shimadzu UV-1800).

### Determination of swelling

In a wire basket, a tablet from each batch (without drug) was selected and placed with its noted weight into 250 mL simulated gastric fluid (pH 1.2) the basket was dipped at 37°C for a period of 12 hours. At the end of 12-hour period, the basket was taken out from the fluid. The surface water was removed with tissue paper and the basket including the tablet was weighed in an electronic balance. Swelling index by the tablet was calculated using the following formula:

W_E_= [(W_1_-W_0_)/W_0_]*100

Where WE are the swelling index (%), W0 is the dry weight of tablet plus basket and W1 is the weight of wet tablet plus basket after removal from the medium.^16^ Analysis was carried out in triplicate.

### Dissolution study

USP type-II (Paddle) apparatus was used for dissolution study, 0.1 M hydrochloric acid as dissolution medium at 75 rpm. The apparatus was maintained at 37°C ± 0.5°C. At the regular time of intervals (1, 2, 3, 4, 5, 6, 8, 12 hours) samples was withdrawn and replaced with fresh 0.1 M hydrochloric acid media. Cumulative % drug release was analyzed at 313 nm by UV-Visible spectrophotometer. Analysis was carried out in sextuplet.

### Scanning electron microscopy study

Scanning electron microscopy photographs of the matrix tablets were taken at the end of 12 hr of dissolution.

### Ex vivo mucoadhesion time

The *ex vivo* mucoadhesion test for the mucoadhesive tablet was performed using goat stomach mucosa. A fresh goat stomach mucosa was collected from a local slaughter shop (Anantapuramu, Andhra Pradesh, India) within an hour of excision and was taken in the laboratory in cold normal saline. The collected stomach mucosa was soaked in simulated gastric fluid (SGF, pH 1.2) for 2 minutes after washing with distilled water and normal saline, using cyanoacrylate glue it was fixed to the inner-side-wall of a 250 mL beaker. By the application of light force by fingertip for the 60 seconds the tablet was pasted on to the mucosal surface, previously wetted with one drop of SGF. Simulated gastric fluid (pH 1.2) of 200 mL quantity was added to the beaker, maintained at 37°C and to simulate the peristaltic movement it was stirred at 50 rpm for a period of 10 hours. The time taken by the tablet for complete erosion or detachment from the mucosal surface was considered as an indicator of *ex vivo* mucoadhesion potential.^17^ Analysis was carried out in triplicate.

The risk assessment of the formulation variables was updated based on the results of the formulation development studies.

### Defining design space

ANOVA generated by Sigma Tech software version (version 3.1) was used to study the effect of independent variables on dependent variables and to generate space based on contour plots.

### Defining control strategy

The controls can include parameters and attributes: Drug substance, drug-product materials, and components, facility and equipment operating conditions, in-process controls, finished-product specifications, the associated methods. Based on the QbD approach a cost-effective formula should be selected with minimal trials.

### Stability studies

Tablets of the optimized formulation were filled in high density polyethylene containers to carry out stability studies at 40° C ±2°C /75% ± 5% relative humidity for 6 months. The optimized formulation was evaluated for hardness and *in vitro* drug release after 6 months.

## Results and Discussion

Organoleptic properties showed that the rosiglitazone maleate was a white, odorless and colorless powder. The melting point of the rosiglitazone maleate was found to be 134°C. Based on physicochemical characteristics, *in vitro* dissolution, pharmacokinetic and clinical characteristics QTPP was carried out on rosiglitazone maleate for the preparation of the desired formulation, needed in the final product. The targets that were majorly opted to formulate an extended-release tablet equivalent to or better than reference listed drug was to extend the drug release in the upper layer of stomach for 12 hours, absorption in the stomach by mucoadhesion. On the basis of safety and efficacy of the formulation i.e. patient acceptability and compliance, desired bioavailability CQAs were identified (Table 1). FT-IR studies confirmed that the drug was compatible with the selected excipients without any interactions. DSC thermograms of pure drug and optimized formulation were obtained at 131.4 and 130.6°C. As there was no major shift in the melting point, it confirmed that there was no interaction which may affect the pharmacotechnical properties of the formulation.

**Table 1:** CQA’s for rosiglitazone maleate extended release tablet

**Drug product**	**Target**	**Is this a CQA**	**Justification**
Physical attributes	Appearance Color and sahape (acceptable by the patient) Odor	No	It is set for patient acceptability and it is not linked directly to safety and efficacy. Should be free from Unpleasant odor.
	Size	No	Set for ease of swallowing and compliance.
	Weight variation (± 5 % of 250 mg)	No	It is a potent drug. For potent drug Assay should be performed rather than weight variation.
	Hardness (>5 kg/cm^2^) Friability (NMT 1.0% w/w)	Yes No	May affect disintegration which may be indirectly related to the release of the drug. Extended release product will have sufficient hardness. Hence the chances of friability is less.
Drug content	> 98% w/w of label claim	Yes	Critical parameter which may affect safety and efficacy without desired strength. It should be performed through out the process
Dissolution	Extended and soluble in 0.1 M HCl	Yes	Failure to meet the dissolution specification can impact bioavailability. Both formulation and process variables affect the dissolution profile. This CQA will be investigated throughout formulation and process development
Mucoadhesion time	Adhesion to the stomach by use of mucoadhesive polymers	Yes	Critical parameter failure to exhibit adhesion to the stomach results in variation of bioavailability.

Parameters like assay, hardness, dissolution and mucoadhesion time were studied on selected polymers (Carbopol 934P, SCMC) using FMEA method which may impact effect the quality and safety of required formulation. Risk assessment was made for selected formulation variables in which assay with low risk and hardness, dissolution, mucoadhesion time with high risks. From the risk assessment, initially Carbopol 934P, SCMC and ethyl cellulose were selected as polymers to study their effect on the formulation. It was found that ethyl cellulose a hydrophobic polymer could sustain the release, with required hardness but could not match with the extended release criteria (NLT 80 % drug release in 12 hours). However the preliminary studies with other selected polymers exhibited required criteria. Hence they were selected for studies (DoE). Carbopol 934P, SCMC were selected as independent variables. The quantities of mucoadhesive polymers that were to be used in the formulation were selected by referring *Handbook of Pharmaceutical Excipients* and by the experimentation done by previous authors. In the present study 2^2^ factorial design with four replicates was selected and further extended to central composite design since the 95% confident levels of curvature effect was positive for hardness and drug release at 12 h, negative for mucoadhesion time (Table 2).

**Table 2:** Experimental design of tablets

**Factor No.**	**Factor**	**Units**	**Levels**				
			**(-2)**	**(-1)**	**(0)**	**(+1)**	**(+2)**
1	Carbopol 934P	mg	70.8	79.6	88.4	97.2	106
2	SCMC	mg	44.5	66.7	89.05	111.3	133.6

SCMC, sodium carboxymethyl cellulose.

Precompression parameters indicated that the prepared blend had good free-flowing property. Compressibility index (CI), Hausner’s ratio and Angle of repose were found to be in the range of 2.27% to 13.2%, 1.02 to 1.11 and 20.58 to 26.69° respectively.

Tablets were compressed by direct compression method and evaluated for post compression parameters which were in compliance with I.P. The percentage drug content for the compressed tablets was in the range of 92.4-103.9 % within the acceptable limits. Swelling studies showed that batch F8 (120.2 %) (g/g) and F5 (90.6 %) (g/g) showed highest and lowest water uptake respectively, among the batches containing higher quantities of Carbopol 934P showed higher swelling index due to the formation of more branched bulky network matrix by the physical entrapment of more water and due to its high hydrophilic property. Hardness, drug release at 12 hours and *ex vivo* mucoadhesion time for all the formulations were found in the range of 6.4 to 7.5 kg/cm^2^, 88.1 to 102.1 %, 4 to 8 hours respectively.

All the responses were analyzed and found that X_1_ was highest with SS ratio of (97.304%), (94.838%) and (48.207%) respectively. A positive sign of the coefficient (0.1417) indicated linear effect on hardness i.e. increased hardness with increase in the concentration of Carbopol 934P, a negative sign of the coefficient (– 3.075) indicated nonlinear effect for drug release at 12 h i.e. as the concentration of Carbopol 934P increased the drug release extended and a positive sign of the coefficient (0.725) indicated linear effect for *ex vivo* mucoadhesion time i.e. increased time with increased concentration of Carbopol 934P. R^2^ model found to be significant hence this model has been used for predictions. Whereas an increase in the concentration of SCMC reduced hardness, mucoadhesion time and showed faster drug release at higher concentration levels (Table 3). The scanning electron microscopy image of the dry tablet showed tough network between particles. On hydration the surfaces of the tablet formed porous film because of the formation of the gel due to polymer relaxation.

**Table 3:** Effect of polymers on responses

**Dependent variable**	**Equation**	**R** ^ 2 ^	**Obtained F-value** ( ***P*** ** < 0.05)**	**Critical F-value** ( ***P*** ** < 0.05)**
Hardness	Y_1_ = 6.078 + 0.1417 X_1_ – 0.048 X_2_ – 0.025 X_1_X_2_ + 0.0667 X_1_^2^ – 0.0333 X_2_^2^	0.989	6.59	4.95
Drug release at 12 h	Y_2_ = 97.0778 – 3.075 X_1_ + 2.3583 X_2_ – 0.775 X_1_X_2_ – 1.9083 X_1_^2^ – 1.0333 X_2_^2^	0.972	5.79	
*Ex vivo* mucoadhesion time	Y_3_ = 5.0861 + 0.725 X_1_ – 0.2417 X_2_ + 0.275 X_1_X_2_ + 0.2792 X_1_^2^ – 0.0958 X_2_^2^	0.985	10.1	

F value, Fisher’s value; R, regression value; *P*, probability.

**Figure 1:**
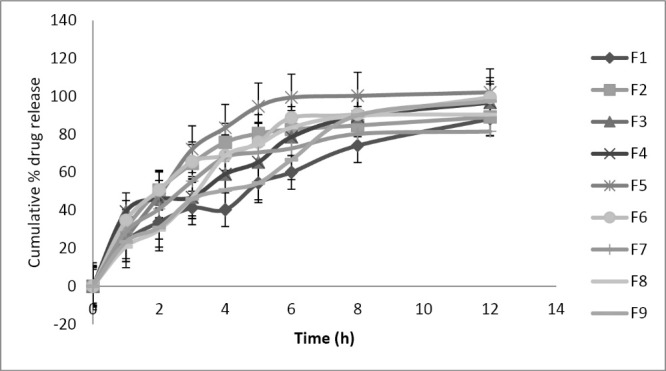
*In vitro* drug release of rosiglitazone maleate from formulations (F1-F9). Values are expressed as the mean ± SD; n = 6.

Drug release for all the formulations could not meet the extended release criteria. F7 batch could control the burst release i.e., NMT 30% release in 2 hours but all other formulations could not satisfy the burst release showed more than 30 % in 2 hours. This might be due to a higher amount of SCMC. Whereas all the formulations satisfied NLT 80% release in 12 hours but the batch F7 showed retarded drug release (81.5 %) due to the presence of higher concentration of Carbopol 934 P (+2) and midlevel concentration of SCMC (0) (Figure 1). The presence of hydrophilic carboxy groups in Carbopol 934P and SCMC might have formed strong hydrogen bonding, that lead to stronger cross-linking for retarding the drug release and extending the mucoadhesion.

Since the relationship between responses Vs factors was nonlinear as shown by Sigma Tech software, the Central composite design has been applied. A significant effect was identified by ANOVA, at *P *< 0.05 the model showed the significant effect as the critical F-value is smaller than the obtained F values. So it can be concluded that the obtained F value is likely to occur by chance with a *P *< 0.05 i.e. indicates significance at that level of probability. R^2^ value of response quadratic model was found to be greater than 0.70 suggesting that this model is reliable for all CQAs. Hence used to establish predictions and contours/design space for developing the Robust method (Table 4).

**Table 4:** ANOVA table

**Source of variance**	**SS**	**df**	**MS**	**Error variance**	**Standard deviation**	**Curvature effect**
**ANOVA table (Hardness)**						
Model	0.9275	3	0.3092	0.0092	0.0957	0.15
Error	0.0	4	0.0			
Total	0.9275	7				
95 % confident level of curvature effect: 0.0654 to 0.3654; Significant						
**ANOVA table (Drug release at 12 h)**						
Model	52.407	3	17.4692	0.74	0.8602	8.975
Error	0.0	4	0.0			
Total	52.407	7				
95 % confident level of curvature effect: 7.0401 to 10.909; Significant						
**ANOVA table (** ***ex vivo*** **mucoadhesion time)**						
Model	0.6275	3	0.2092	0.0292	0.1708	1.4
Error	0.0	4	0.0			
Total	0.6275	7				
95 % confident level of curvature effect: – 1.7841 to – 1.0159; Significant						

ANOVA, analysis of variance; SS, sum of squares; df, degrees of freedom; MS, mean square.

**Figure 2:**
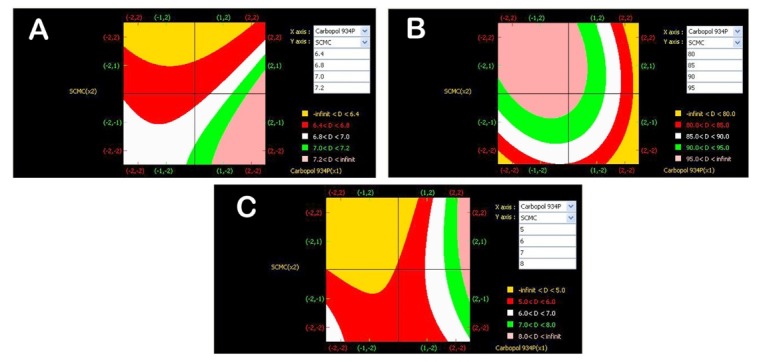
Contour plots for rosiglitazone maleate tablets (A) Hardness (B) *Invitro* drug release (C) *Ex vivo* mucoadhesion time.

The risk was reduced to an acceptable level after defining, executing the experimental studies, establishing scientific knowledge and understanding that allowed appropriate control for the development, implementation. Polymer levels have been selected and optimized to give hardness, drug release, mucoadhesion as per QTTP. Based on the results, formulation variables were updated for risk assessment. Design space was constructed and shown in Figure 2 which could be the operating ranges. Design space for Carbopol 934P and SCMC was found to be 70.8 mg to 106 mg and 44.5 mg to 89.05 mg respectively to get desired responses that match QTPP. Risk assessment, experimental design, literature, and prior knowledge contributed in defining the design space. An optimized formulation was selected based on contour plots, simulation as predicted by the software and knowledge from *in vitro* drug release studies conducted for the formulations F1 to F9. Based on the predicted values the formulation was prepared and evaluated for all the parameters. It was concluded that predicted values (7.4 kg/cm^2^ hardness, 83.4% drug release at 12 hours, 7.6 hours*ex vivo* mucoadhesion time) were closer to experimental values (7.3 kg/cm^2^ hardness, 85.7% drug release at 12 hours, 7.7 hours*ex vivo* mucoadhesion time) which were confirmed by calculating standard error less than 1.15 i.e. at the selected composition of Carbopol 934P at 106 mg/tab and SCMC at 66.7 mg/tab. Drug release kinetics for optimized formulation followed first order release with Higuchi diffusion and Fickian diffusion. Marketed extended-release tablets of rosiglitazone maleate was unavailable in the market, hence the conventional tablets of the brand (Rositus tablet) marketed by Aretaeus Pharma were purchased and conducted *in vitro* dissolution study. Execution of an operating plan, including an appropriate control strategy and appropriate process monitoring, helps in successful development and improvement of product and process performance.

Stability study of optimized formulation showed that tablets were stable without any significant changes and were within specifications.

## Conclusion

QbD is an efficient tool that gives the product with desired quality and characteristics based on cost-effective procedures by a thorough understanding of formulation and process variables through science and risk-based approach. The present study has shown that application of QbD in the development and optimization of rosiglitazone maleate mucoadhesive extended-release tablets were formulated which extended the drug release for 12 hours by mucoadhesion thereby reducing the frequency of dosing by improved patient compliance. Further *in vivo* studies should be performed to establish pharmacokinetic and pharmacodynamic parameters on the optimized rosiglitazone maleate mucoadhesive extended-release tablets.

## Ethical Issues

Not applicable.

## Conflict of Interest

There is no conflict of interest to declare.

## Acknowledgments

The authors thank Principal Raghavendra Institute of Pharmaceutical Education and Research for providing necessary facilities to carry out the research work and also thank Connexios Life Sciences Pvt Ltd for providing Rosiglitazone maleate drug sample.
